# Vaccination against COVID-19 and inequalities – Avoiding making a bad situation worse

**DOI:** 10.1016/j.puhip.2021.100101

**Published:** 2021-03-03

**Authors:** Michelle Black, John Ford, Andrew Lee

**Affiliations:** School of Health and Related Research (ScHARR), The University of Sheffield, Sheffield, UK; School of Clinical Medicine, Cambridge University, Cambridge, UK; School of Health and Related Research (ScHARR), The University of Sheffield, Sheffield, UK

The implementation of COVID-19 vaccination programmes is actively being pursued by many countries worldwide. For them, successful vaccination programmes could pave the way for societies to safely emerge from the pandemic. Time will tell just how effective these vaccination programmes will be in reducing transmission, as well as whether the emergence of vaccine-resistant mutations will occur that may render them less effective.

The UK was the first country to start vaccinating its population and its vaccination strategy and pace of delivery is to be commended. The vaccine has been targeted at nine priority groups which represent 99% of preventable deaths from COVID-19 [1]. In addition, the UK strategy recommends that attention is given to ‘mitigating health inequalities, such as might occur in relation to access to healthcare and ethnicity’ [1]. It is hoped that this will avoid further compounding of the substantial pre-existing health inequalities which have worsened during the pandemic [2,3].

Analysis of the initial weeks of the vaccination programme in England highlights emerging inequalities in vaccine coverage with lower rates in ethnic minorities, people living in areas of higher deprivation, and those with severe mental illness or learning disabilities [4]. This is concerning but unsurprising as previous research has shown that universal interventions can increase inequality because of the differential uptake between communities [5,6].

There is a moral argument not to worsen inequalities through inequitable vaccine distribution, but what can be done? Open and transparent decision making, the provision of comprehensive and accessible data, bold action, as well as efforts to improve public trust and engagement will help. The rationale for vaccine prioritisation decisions has to be clearly articulated in order to counter political pressure from various bodies lobbying to change vaccine priority rankings, such as trade unions, advocacy groups and political parties. It is important that the needs of all population groups are considered, not just those who shout loudest, to avoid exacerbating inequalities by prioritising lower risk groups over those at higher risk of adverse health outcomes.

Transparent, robust data on the distribution of the vaccine for each nation is urgently needed. National UK data are published on the total number of people vaccinated daily, with data in England including a breakdown by age and ethnicity. However, data is not currently published on vaccination rates in each priority group, vaccine uptake by deprivation, proportion of vaccinations declined, nor on the reasons for vaccine refusal. The provision of comprehensive vaccine coverage data is vital so that any access issues can be identified [1]. In turn, policy-makers, practitioners and researchers can develop early solutions to address these issues.

Bold action is needed on health inequalities. Evidence and history show that specific policy goals, with careful alignment of targets, as well as the allocation of resources proportionate to need, are required [7]. Policy to address inequalities has to be specific, adequately resourced, and take into account the context of imbalances in resources, funding, workforce and investment across communities that may have accumulated over decades. Action on inequalities have to be undertaken at the national, health system, organisation and individual level. National policy and health systems need to ensure that vaccine distribution and funding to deliver the vaccines are proportionately weighted to the areas with greatest disadvantage. Using a data-driven approach, organisations such as primary care providers can identify and target patients who require additional support to access the vaccine. Going the extra mile to vaccinate underserved patients takes time and resources.

Public trust and engagement has been essential in encouraging people to abide by public health instructions and restrictions. They are equally important in ensuring the success of vaccination initiatives. However, trust across certain communities within society may have been eroded through the reinforcement of power hierarchies, where instead of working in partnership, one section of society enforces life-changing policies on another, such as through punitive law enforcement. For example, socioeconomically disadvantaged communities that have suffered from years of austerity following a recession are now being told what they must do for the benefit of wider society. Regaining trust involves working collaboratively with communities, treating them as equal partners and making efforts to understand the rationale underpinning their vaccine choices.

In the UK, equality and health inequalities impact assessments are currently being used by some local health systems to mitigate the inequalities that may arise from the vaccine delivery programme. We will have to wait to see if these have been effective. If done meaningfully, at a strategic and operational level, early in decision making and focused on the redistribution of resources, they may make a substantial difference. However, if they become a bureaucratic process that focuses on blunt targets and small operational changes to the vaccine delivery, they will only ever have a marginal effect.

As the virus adapts and evolves, so too will the strategy to defeat it. Now, more than ever we need to consider the impact on inequalities of the decisions we make. Vaccine strategies will be considerably strengthened if we work in partnership to ensure those who need it most benefit from it. This is true at both the national and global level. The collective effort of countries working towards equitable vaccine access, rather than vaccine nationalism, will help reduce inequalities within and between countries. In turn this will reduce our shared vulnerability to this pandemic.
